# Successful Management of a Foreign Body Impacted in the Hard Palate: A Case Report From a Rural Setting

**DOI:** 10.1002/ccr3.71778

**Published:** 2026-01-02

**Authors:** Shishir Pokhrel, Sudesh Jang Thapa, Eliza Karki, Sabnam Sangraula, Naveen Gautam

**Affiliations:** ^1^ Department of Otorhinolaryngology Rapti Provincial Hospital Tulsipur Nepal; ^2^ Rapti Lifecare Hospital Tulsipur Nepal; ^3^ Department of Psychiatry Tulsipur Nepal; ^4^ Department of Anesthesiology Tulsipur Nepal; ^5^ Rapti Provincial Hospital Tulsipur Nepal

**Keywords:** anesthesia, foreign body impaction, hard palate, pediatric, rural setting

## Abstract

Foreign body ingestion and impaction in children pose significant challenges, particularly in atypical locations like the hard palate. This case report details the successful management of a doll's eye embedded in the hard palate of an 18‐month‐old female child in a rural setting, highlighting the importance of clinical acumen, resourcefulness, and prompt intervention. Despite limited specialized care access, the foreign body was safely extracted under intravenous anesthesia using basic surgical facilities. This case underscores the risks associated with foreign body impaction in young children and emphasizes the need for preventive strategies and parental education.

## Key Clinical Message

Successful management of hard palate foreign body impaction in a resource‐limited setting is achievable with prompt recognition, appropriate anesthesia, and basic surgical facilities. This case emphasizes the importance of clinical vigilance and preventive measures to reduce the incidence of such occurrences in young children.

## 1 Introduction

Foreign body ingestion and impaction, which affect up to 75% of cases in children aged 6 months to 3 years, present significant diagnostic and therapeutic challenges in pediatric care [1, 2]. While the esophagus and airway are common sites, hard palate impaction is very rare [3]. Due to its anatomical constraints and potential complications, it poses unique management difficulties [4]. Delayed intervention can lead to severe outcomes, including infection, airway obstruction, or palatal perforation [4].

This case report details the successful management of a foreign body embedded in the hard palate of an 18‐month‐old female child in a rural setting. It highlights the critical role of clinical acumen, resourcefulness, and prompt intervention, especially where specialized care access is limited. The case emphasizes the risks associated with foreign body impaction in young children and underscores the importance of preventive strategies and parental education in reducing incidence rates.

## 2 Case History/Examination

An 18‐month‐old female child (weight: 11.2 kg, height: 82 cm) presented to the emergency department of a rural hospital with a foreign body impacted in the hard palate. The child's mother reported witnessing the incident 30 min prior, when the patient was playing with a doll and suddenly brought her hand to her mouth.

**FIGURE 1:**
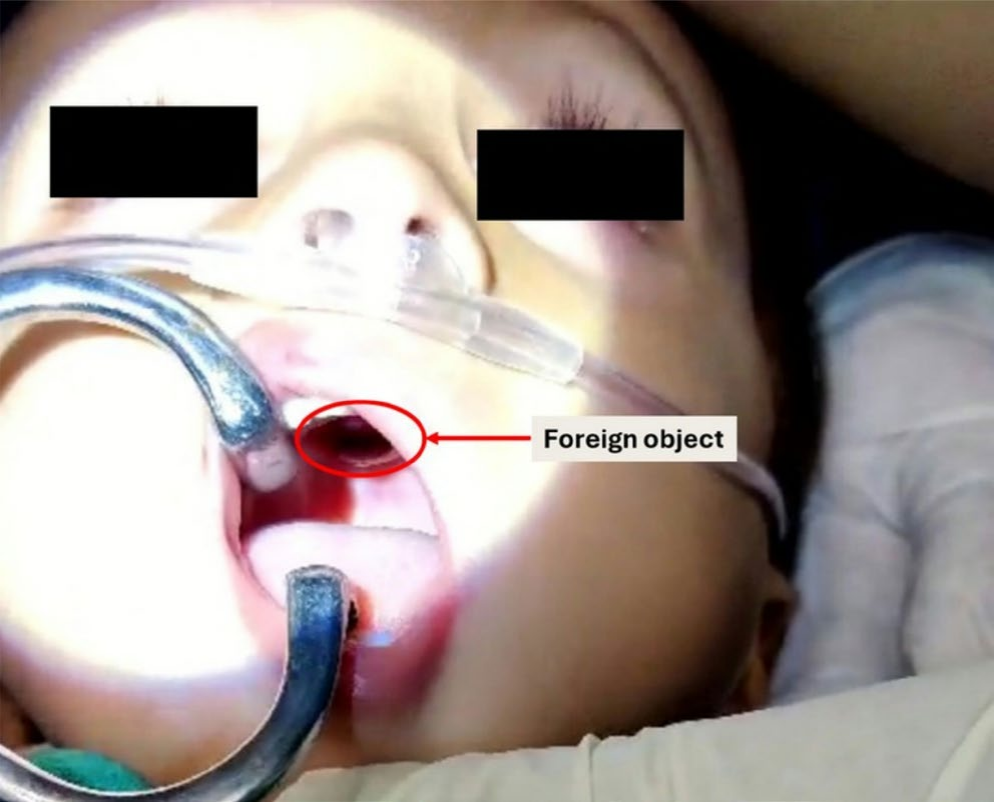
Clinical photograph showing the foreign body embedded in the hard palate.

On examination, vital signs were stable (heart rate: 110 bpm, respiratory rate: 24/min, temperature: 37.0°C, SpO_2_: 99% on room air). Oral inspection revealed a cylindrical plastic object, identified as a doll's eye (diameter: 5 mm), firmly embedded in the anterior hard palate, 1.5 cm posterior to the incisive papilla (Figure 1). The surrounding mucosa showed no erythema, edema, or active hemorrhage.

## 3 Methods

**FIGURE 2:**
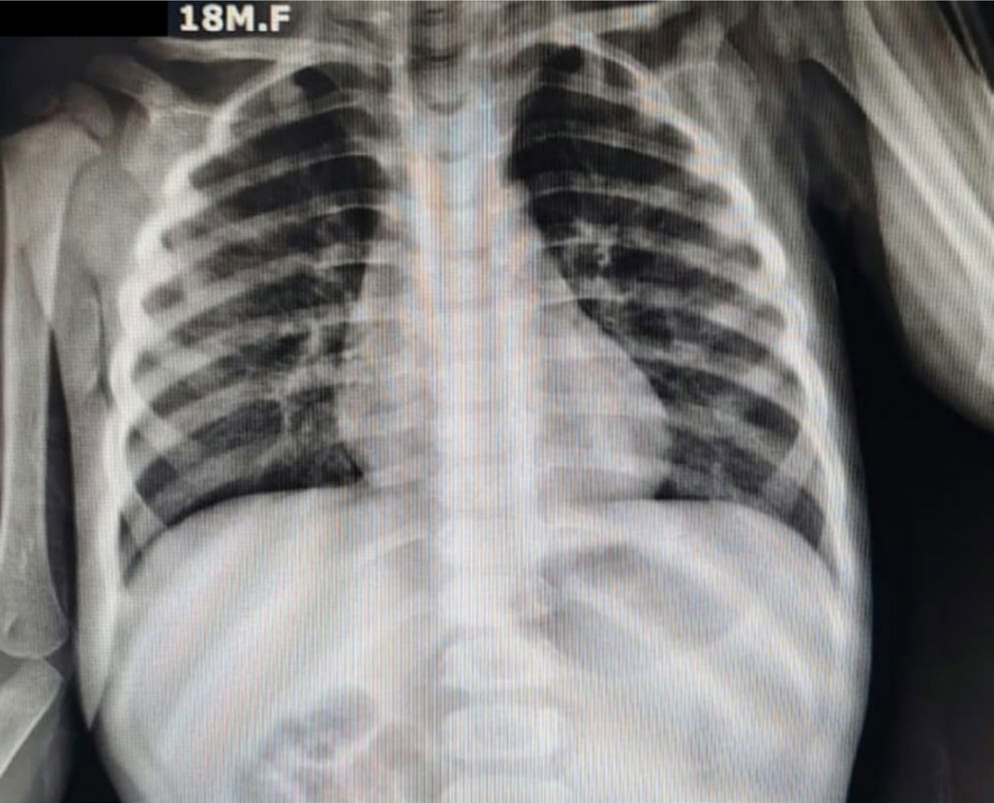
Normal chest X‐ray of the child showing no features of aspiration.

Initial attempts at manual extraction using Magill forceps were unsuccessful. Preoperative investigations included complete blood count, coagulation profile, and serum electrolytes, all within normal ranges (Table 1). A chest X‐ray was performed, showing no evidence of aspiration or airway obstruction (Figure 2).

**TABLE 1:** Preoperative investigations and findings.

Investigation	Result	Normal Range
**Complete blood count (CBC)**		
‐ Hemoglobin	11.5 g/dL	10.5–13.5 g/dL
‐ White blood cell count	9500/μL	6000‐17,000/μL
‐ Platelet count	280,000/μL	150,000‐450,000/μL
**Coagulation profile**		
‐ Prothrombin time (PT)	12 s	11–13 s
‐ Activated partial thromboplastin time (aPTT)	30 s	25–35 s
‐ International normalized ratio (INR)	1	0.8–1.2
Chest X‐ray	No evidence of aspiration or airway obstruction (Figure 2)	N/A
Blood glucose	85 mg/dL	70–100 mg/dL
**Serum electrolytes**		
‐ Sodium	138 mEq/L	135–145 mEq/L
‐ Potassium	4.0 mEq/L	3.5–5.0 mEq/L
‐ Chloride	102 mEq/L	98–107 mEq/L
‐ Bicarbonate	22 mEq/L	22–26 mEq/L

**FIGURE 3:**
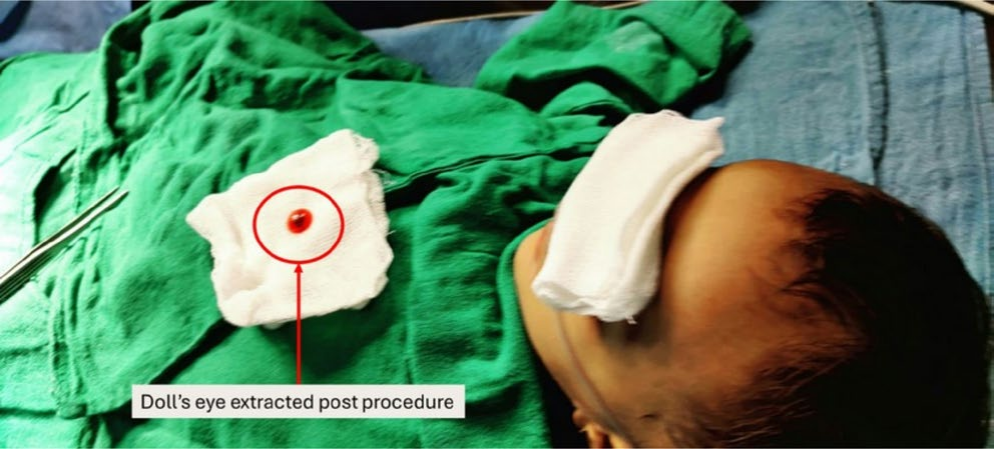
Photograph showing extracted foreign object postprocedure.

The patient was transferred to the operating theater. Anesthesia was induced with propofol (2.5 mg/kg IV) and ketamine (1 mg/kg IV). Glycopyrrolate (5 μg/kg IV) was administered for antisialagogue effect. A Dingman mouth gag provided optimal visualization. Using a 5‐in. curved hemostatic forceps, the foreign body was extracted with a gentle rotational movement. The total procedure time was 7 min. Minimal bleeding was controlled with direct pressure for 30 s. Postextraction examination revealed an intact mucosal surface. The extracted foreign body, likely the doll's eye, is shown in (Figure 3).

## 4 Conclusion and Results

The patient remained NPO for 4 h postprocedure. Oral intake was gradually reintroduced, starting with clear liquids at 4 h and advancing to a soft diet at 8 h postprocedure. Prophylactic amoxicillin (40 mg/kg/day in 3 divided doses) was administered for 5 days. The child was observed for 48 h postprocedure. No complications were noted during this period. She was discharged with instructions for routine follow‐up and parental education on foreign body prevention. Follow‐up examinations at 1 week and 1‐month post‐procedure showed complete healing of the palatal mucosa with no residual defect or functional impairment.

## 5 Discussion

Foreign body ingestion and impaction in the pediatric population represent a significant challenge in emergency medicine, most commonly occurring in children aged 6 months to 4 years [5]. While most cases involve the gastrointestinal tract, atypical locations such as the hard palate warrant particular attention due to their unique anatomical and clinical implications [3]. This case of a doll's eye impacted in the hard palate of an 18‐month‐old female child exemplifies the complexities inherent in managing such presentations, particularly in resource‐limited settings.

The rarity of hard palate foreign body impaction is underscored by a comprehensive review by Takaoka et al., which identified only 24 cases in the literature over 29 years [4]. The literature documents instances of diverse objects, including artificial nails, plastic fragments, and screw covers [4]. Our case reinforces the critical importance of maintaining a high index of suspicion for foreign body impaction, even in atypical locations, to prevent diagnostic delays and potential complications.

The management approach in this case aligns with current best practices in pediatric foreign body removal [6]. The decision to utilize intravenous anesthesia, employing a combination of propofol and ketamine, facilitated optimal conditions for extraction while minimizing patient distress and the risk of aspiration [6]. This approach is particularly pertinent in the context of hard palate impactions, where patient cooperation and immobility are crucial for safe removal.

The use of glycopyrrolate as an antisialagogue represents a nuanced consideration in airway management during such procedures. By reducing salivary secretions, it enhances visualization and potentially reduces the risk of aspiration, aligning with recommendations from recent pediatric anesthesia protocols [6].

Since the patient showed no signs of distress or other concerning symptoms, further imaging was deemed unnecessary, except for a chest X‐ray to rule out aspiration. The decision to avoid additional radiological imaging was informed by the clear and unobstructed visualization of the foreign body. This approach minimized radiation exposure and expedited management; a consideration particularly relevant in pediatric cases where the ALARA (As Low As Reasonably Achievable) principle is paramount [7]. However, it is essential to note that in cases where the nature or extent of the foreign body is unclear, advanced imaging modalities such as CT may be warranted to guide management decisions [8].

Postoperative antibiotic prophylaxis, administered as amoxicillin, aligns with current guidelines for preventing secondary infection in cases involving mucosal breach or prolonged impaction [6]. However, the optimal duration of prophylaxis remains a subject of debate, with some studies suggesting that a shorter course may be equally effective while minimizing the risk of antimicrobial resistance [9].

The successful outcome in this case underscores the importance of a multidisciplinary approach, even in resource‐limited settings. The availability of basic surgical facilities and anesthesia services proved crucial in facilitating timely and effective intervention. This highlights the need for ongoing efforts to enhance emergency care capabilities in rural healthcare settings, potentially through telemedicine support or periodic specialist outreach programs [10].

From a public health perspective, this case reinforces the critical need for targeted interventions aimed at preventing foreign body ingestion and impaction in young children. Educational initiatives focusing on parental awareness of age‐appropriate toys and proper supervision during play have demonstrated efficacy in reducing incident rates [11]. Implementation of such programs, particularly in rural areas, could significantly mitigate the risk of similar occurrences.

## 6 Conclusion

This case contributes to the limited body of literature on hard palate foreign body impactions in pediatric patients, offering insights into effective management strategies in resource‐constrained settings. It underscores the importance of clinical acumen, appropriate anesthetic management, and a tailored approach to each unique presentation. Future research directions should focus on optimizing diagnostic algorithms for atypical foreign body locations and refining evidence‐based guidelines for antibiotic prophylaxis in such cases.

## Author Contributions

**Shishir Pokhrel:** conceptualization, data curation, investigation, methodology, project administration, resources, supervision, writing – original draft, writing – review and editing. **Sudesh Jang Thapa:** conceptualization, investigation, methodology, visualization, writing – original draft. **Eliza Karki:** conceptualization, investigation, methodology, supervision, validation, writing – original draft. **Sabnam Sangraula:** conceptualization, formal analysis, methodology, project administration, supervision, writing – original draft. **Naveen Gautam:** conceptualization, investigation, methodology, validation, writing – review and editing.

## Funding

No external funding was received.

## Consent

Written informed consent was obtained from the patient's parent to publish this report in accordance with the journal's patient consent policy.

## Conflicts of Interest

The authors declare no conflicts of interest.

## Data Availability

Data sharing is not applicable to this article as no datasets were generated or analyzed during the current study.
